# Epstein–Barr virus- and cytomegalovirus-specific immune response in patients with brain cancer

**DOI:** 10.1186/s12967-018-1557-9

**Published:** 2018-07-03

**Authors:** Zhenjiang Liu, Thomas Poiret, Qingda Meng, Martin Rao, Anna von Landenberg, Esther Schoutrop, Davide Valentini, Ernest Dodoo, Inti Peredo-Harvey, Markus Maeurer

**Affiliations:** 1 Department of Laboratory Medicine (LABMED), Karolinska Institutet, Stockholm, Sweden; 2 Sun Yat-sen University Cancer Center, State Key Laboratory of Oncology in South China, Collaborative Innovation Center for Cancer Medicine, Guangzhou, People’s Republic of China; 3 Therapeutic Immunology, Karolinska University Hospital Huddinge, F79, LabMed, Hälsovägen, 14186 Huddinge, Sweden; 4 Karolinska Institutet, Stockholm, Sweden

**Keywords:** Brain tumor, Pancreatic cancer, Epstein–Barr virus, Human cytomegalovirus, Cellular immunity

## Abstract

**Background:**

Patients with brain tumor or pancreatic cancer exhibit the poorest prognosis, while immune fitness and cellular immune exhaustion impacts their survival immensely. This work identifies differences in the immune reactivity to the common human pathogens cytomegalovirus (CMV) and Epstein–Barr virus (EBV) between patients with brain tumor in comparison to those with pancreatic cancer and healthy individuals.

**Methods:**

We characterized the humoral and cellular immune responses of patients with brain tumor or pancreatic cancer to cytomegalovirus structural protein pp65 (CMV-pp65) as well as Epstein–Barr nuclear antigen-1 (EBNA-1) by whole-blood assay and ELISA.

**Results:**

Anti-CMV-pp65 plasma immunoglobulin gamma (IgG) titers were significantly lower in patients with brain tumor compared to healthy donors and patients with pancreatic cancer. Among the responding patients with GBM, those with a weak anti-CMV IgG response also had a decreased median overall survival (p = 0.017, 667 vs 419 days) while patients with brain tumor showed a generally suppressed anti-CMV immune-reactivity. Patients with brain tumor exhibited a significantly lower interferon gamma (IFNγ) response to EBNA-1 and CMV-pp65 compared to patients with pancreatic cancer or healthy donors. This antigen-specific response was further amplified in patients with brain tumor upon conditioning of whole blood with IL-2/IL-15/IL-21. Exclusively in this setting, among the responding patients with GBM, those exhibiting a EBV-specific cellular immune response above the median also displayed an increased median overall survival pattern compared to weak responders (753 vs 370 days, p < 0.001).

**Conclusions:**

This report provides (i) a fast and easy assay using common viral antigens and cytokine stimulation to screen for immune fitness/exhaustion of patients with brain tumor in comparison to pancreatic cancer and healthy individuals and (ii) EBV/CMV-induced IFNγ production as a potential marker of survival in patients with brain tumor.

**Electronic supplementary material:**

The online version of this article (10.1186/s12967-018-1557-9) contains supplementary material, which is available to authorized users.

## Background

Human malignant glioma (brain tumor) and pancreatic cancer are characterized by poor prognosis following diagnosis. Patients with pancreatic cancer have a 5-year survival rate of 5% [1], while the situation is worse for patients diagnosed with glioblastoma multiforme (GBM), the most malignant form of glioma, whose 5-year survival rate is frequently below 3% despite treatment [1–3]. Studies have revealed that the tumor microenvironment in patients with either cancer diagnosis is dysfunctional and immunosuppressive in nature, characterized by the low number of tumor infiltrating lymphocytes (TILs), dysfunctional T-cell receptors (TCR), T-helper 2 (Th2) skewed cytokine and generally low tumor immunogenicity [2, 4, 5]. It is therefore plausible to assume that these underlying biological factors may inevitably have an observable effect on immune responses to common infectious agents, including Epstein–Barr virus (EBV) and cytomegalovirus (CMV).

CMV- or EBV-associated disease mostly occurs in individuals with a compromised immune system arising from transplantation, human immunodeficiency virus co-infection (HIV), autoimmunity or cancer to list a few [6]. EBV and to a lesser extent CMV are implicated in cancer owing to their onco-modulatory properties. Nasopharyngeal carcinoma (NPC) and post-transplant lymphoproliferative disorder (PTLD) are attributed to EBV-driven pathogenesis. Furthermore, active EBV can perpetrate acute clinical pancreatitis, where reduced numbers of CD57+ CD8 T cells have been observed, indicating hampered cell-mediated immune responses in conjunction with chronic immune activation and reduced responsiveness to cognate epitopes [7, 8]. Interferon gamma (IFNγ) and tumor necrosis factor alpha (TNFα) production by CD4+ T helper 1 (Th1) targeting EBV nuclear antigen 1 (EBNA-1) in patients with breast cancer harboring the latent form of EBV correlated with better clinical outcome compared to those infected with the replicative form of the virus [9]. No direct association of EBV infection in brain tumor or pancreatic cancer has been shown to date although its implication in glioma has drawn recent attention [10]. Although initial studies link anticancer responses potentially orchestrated by EBV-specific T cells, this hypothesis requires formal testing.

CMV-derived components such as viral DNA and protein antigens in GBM tumors have been long debated and remain controversial [11]. CMV-positive patients with GBM receiving valganciclovir adjunctively to standard chemotherapy showed overall improved survival after antiviral therapy [12], yet other groups have not observed any evidence of increased CMV DNA load in the tumor [13]. Interestingly, human cytotoxic T cells specific for CMV structural protein pp65 (CMV-pp65) can recognize and kill human primary GBM as well as leukemic cells [14, 15], while a CMV-based vaccine candidate facilitated enhanced survival among patients with GBM in a recent clinical trial [16].

Several reports have described the humoral response to CMV and EBV in association with brain tumor [17–19]. However, to the best of our knowledge, specific IFNγ production to EBV and CMV in patients with pancreatic cancer or brain tumor remains unexplored. CMV-pp65 is a strong TCR agonist that is widely used to gauge the immune competence of individuals, while EBNA-1 is associated with latent EBV infection [20], and contains epitopes capable of inducing strong CD4+ T-cell responses and antibody production.

While the specific IFNγ production to tumor associated antigen (TAA) would highlight the exclusive response to cancer antigen, IFNγ response to common viral antigens in Sweden such as CMV-pp65 and EBNA-1 (prevalence above 80%) may reflect general immunological balance versus immune exhaustion [21, 22]. We reported here the anti-CMV/EBV humoral and cellular immune response profile of patients with brain tumor in comparison to those with pancreatic cancer and healthy individuals (HD). The results of this study are expected to contribute to developing CMV/EBV-directed immune reactivity as valuable biomarkers of immune fitness in patients with advanced cancer.

## Methods

### Patient characteristics/cohort description

A total of 374 patients with brain (n = 314) or pancreatic cancer (n = 60) registered at the Karolinska University Hospital, Stockholm were recruited for this study following informed consent (diary numbers: 2013/576-31 and 2013/977-31). Patients with brain tumor presented with the following diagnoses: glioblastoma multiforme (GBM), astrocytoma (A), oligoastrocytoma/oligodendroglioma (OA/OD) or metastases (M). Patients with brain tumor received corticosteroids (betamethasone) prior to blood sampling as described earlier [23]. Blood samples were collected in heparin-containing vacutainer tubes on the day of surgery prior to initiation of anesthesia and processed in the laboratory within 24 h. Plasma was obtained after centrifugation and stored at − 20 °C for later analysis. None of the participating patients had undergone surgery or started chemotherapy at sample collection. The clinical characteristics of the study cohort are summarized in Table 1. Samples from age- and sex-matched healthy donors (HD) recruited for a previous study (DN 2009/1183-3) were used as baseline controls (n = 244) [24].

**Table 1:** Patients information

Patient characteristics	Brain tumor				Pancreatic cancer	Healthy donor
	GBM	A	OA/OD	M		
Sample size (N)	185	52	33	44	60	244
Age median (years)	63	34	41	60	72	57
Age range (years)	16–80	19–75	22–62	30–84	42–88	17–87
Gender (M/F) %	66/34	73/27	48/52	46/54	62/38	58/42

Clinical characteristics of cancer patients and healthy individuals participating in the study

*M/F* male/female, *GBM* glioblastoma multiforme, *A* astrocytoma, *OA/OD* oligoastrocytoma/oligodendroglioma, *M* metastatic disease

### Quantitative indirect ELISA for antigen-specific plasma IgG

CMV- and EBV-specific IgG was evaluated by quantitative indirect ELISA previously described [25]. Briefly, in a 96-well ELISA plate, human IgG (Sigma, USA) was used for reference standard in a 7-point serial dilution (1:2 ratio) in duplicates and EBNA-1 and CMV-pp65 whole proteins (CMV-215-C and EBV-271-C, Prospec, Ness-Ziona, Israel) as coating antigens. The plate was incubated for 1 h at 37 °C. After five washes, diluted patient plasma samples were added to the assay plate and incubated for 2 h at 20 °C. After 5 washes, the plate was incubated with a secondary anti-human IgG monoclonal antibody (Alkaline phosphatase-conjugated, 1:1000 dilution, Mabtech, Stockholm, Sweden) for 1 h at 20 °C. Para-nitrophenylphosphate (pNPP, Thermo Fisher Scientific, MA, USA) was then added and incubated for 45 min at 20 °C in the dark and the reaction was stopped by adding 1 N sodium hydroxide (NaOH). The optical density was measured at 405 nm using a Vmax kinetic microplate reader.

### Whole blood assay (WBA) and IFNγ enzyme-linked immunosorbent assay (ELISA)

Whole blood was first diluted at a ratio of 1:1.5 with either RPMI 1640 medium containing l-glutamine (2 mM) with antibiotics (100 IU/ml penicillin and 100 µg/ml streptomycin) (Life Technologies, Carlsbad, USA) alone or supplemented with the following cytokine cocktail: IL-2 (1000 IU/ml), IL-15 (10 ng/ml) and IL-21 (10 ng/ml) (Prospec, Ness-Ziona, Israel). Diluted blood was added in duplicates for each condition to 96-well plates pre-coated with the proteins EBNA-1 or CMV-pp65 (Prospec, Ness-Ziona, Israel) at a final concentration of 1 μg/ml and incubated for 7 days at 37 °C with 5% CO2 as previously described [26, 27]. Antigen-free medium was used as negative control while phytohemagglutinin protein (PHA 5 μg/ml, Sigma Aldrich) was used positive control. Cell culture supernatants were harvested after the incubation period to quantify IFNγ production by sandwich ELISA (Mabtech, Stockholm, Sweden) according to the manufacturer’s instructions. Assay plates were analyzed using a Vmax kinetic microplate reader (Molecular Devices, USA) at 450 nm. After basal IFNγ production (medium only) subtraction, data were reported as absolute cytokine concentration values (pg/ml).

### Data processing and statistical analysis

First, data were reported and analyzed as absolute antigen-specific IFNγ production (pg/ml). To further assess the patients’ ability to respond to the specific viral antigens and analyze between patient groups and healthy donors, we estimated the virus-specific IFNγ production relative to the basal IFNγ production (medium only, no stimulation) by dividing the virus-specific IFNγ production by the basal IFNγ production. This provides the relative IFNγ production (rIFNγ) by circulating lymphocytes directed against CMV-pp65 or EBNA-1. To characterize the impact of the cytokine cocktail (IL-2/IL-15/IL-21) on antigen-specific IFNγ production, the delta (Δ) value of difference was reported as ΔIFNγ production calculated by the antigen-specific IFNγ production with cytokine conditioning subtracted by the antigen-specific IFNγ production without cytokine conditioning. GraphPad Prism 6 software (La Jolla, California, USA) was used for data processing and statistical analysis. The Kruskal–Wallis test followed by Dunn’s post-test was used to compare the different group of patients with controls. Correlation was assessed by linear regression (r^2^ value) after log transformation of the data, 0 values were ascribed a nominal values of 0,1 pg/ml. Patient survival was evaluated via Kaplan–Meier survival analysis with log-rank test over 1168 days after sampling and surgery (post-OP). The patients were classified as either high or low IFNγ producers, or having high/low IgG titers according to the median value measured. *p* value of less than 0.05 was considered significant.

## Results

### Specific IgG response to CMV-pp65 among patients with cancer and healthy donors

**Fig. 1:**
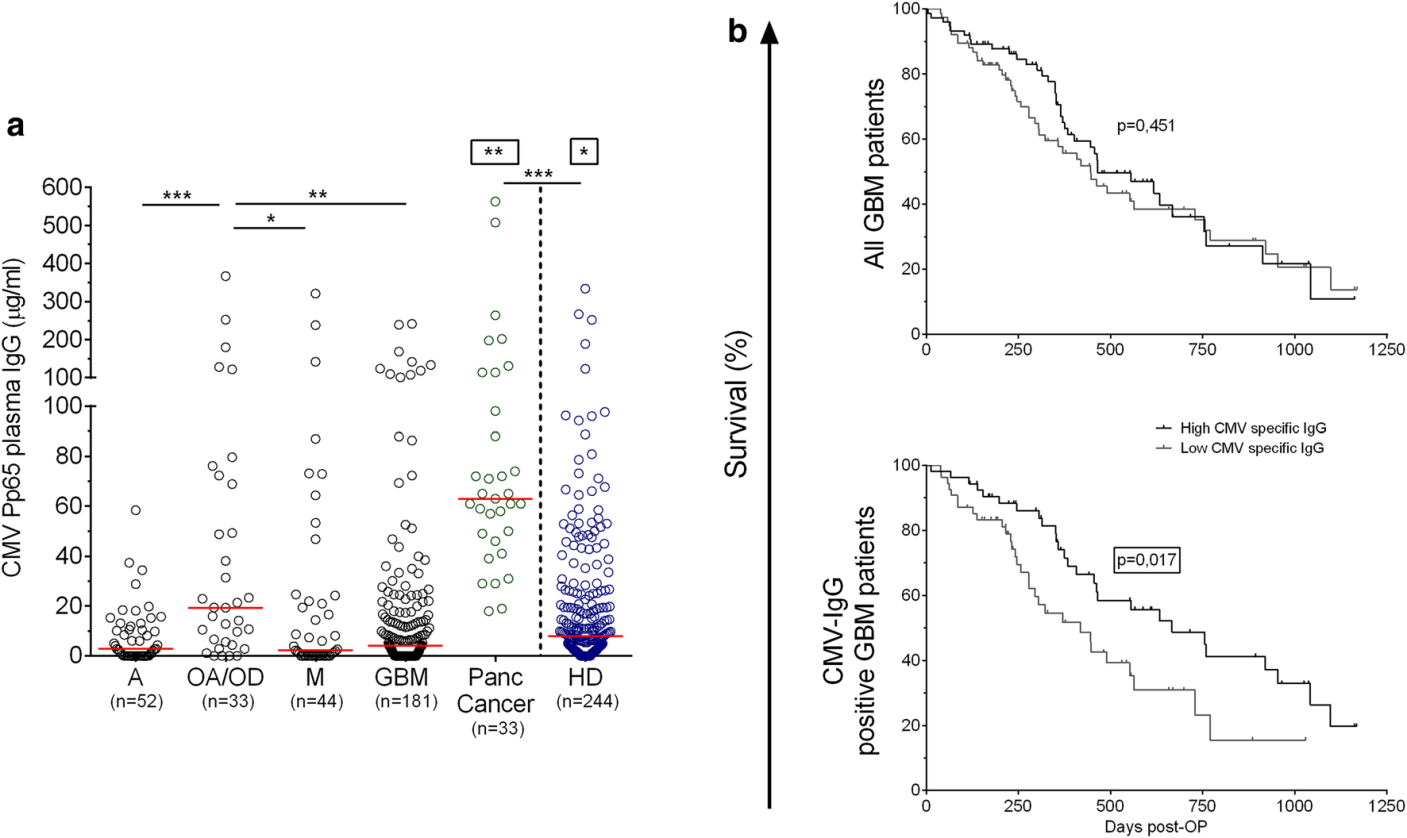
**a** IgG recognition of CMV-pp65 in plasma of healthy donors and patients with brain tumor or pancreatic cancer. Box stars indicate statistical significance of IgG levels in response to CMV-pp65 with all the other groups. Medians are shown for each group when superior to 0. Kruskal–Wallis test followed by Dunn’s post-test was performed to gauge statistical significance *p < 0.05; **p < 0.01; ***p < 0.001. **b** Survival of the patients with GBM based on the CMV-specific IgG levels. Median concentration of CMV-specific IgG levels was used as a cut-off to generate two groups: individual with “high” vs. “low” CMV-specific IgG. Top panel: all GBM patients (n = 151). Bottom panel: GBM patients with detectable anti-CMV IgG (n = 107). *GBM* glioblastoma multiforme, *A* astrocytoma, *OA/OD* oligoastrocytoma/oligodendroglioma, *M* metastatic disease, *Panc Cancer* pancreatic cancer, *HD* healthy donor and *Post-OP* post-operation

Before evaluating the EBV- and CMV-specific cellular immune response of patients with brain cancer, we determined the titers of plasma IgG specific for EBNA-1 or CMV-pp65 using an indirect ELISA method developed in-house. After sex- and age-matching of the groups with healthy donors (HD), we compared plasma IgG recognition of EBNA-1 or CMV-pp65 among patients with brain tumor or pancreatic cancer with that of HD. No statistically significant differences in the recognition of EBNA-1 by plasma IgG were observed (data not shown). However, we did observe a significantly low level of CMV-pp65-specific IgG among patients with brain tumor compared to those with pancreatic cancer (at least p < 0.001, Fig. 1 a). With the exception of patients with oligoastrocytoma/oligodendroglioma (OA/OD) showing higher humoral immune recognition of CMV-pp65 compared to patients with other diagnoses of brain tumor (at least p < 0.05), patients with brain tumor had a significantly low level of CMV-pp65-specific plasma IgG (p < 0.05) compared to healthy individuals (HD). The overall survival of patients with GBM post-OP was not associated with humoral immune responses to CMV-pp65 (465 vs 446 days, Fig. 1 b, top panel). However, among patients with detectable anti-CMV IgG, those with higher titers of antibody exhibited an increased overall median survival than those with lower IgG titers (p = 0.017, 667 vs 419 days, Fig. 1 b, bottom panel).

### IFNγ responses of patients with brain tumor to EBV and CMV antigens

**Fig. 2:**
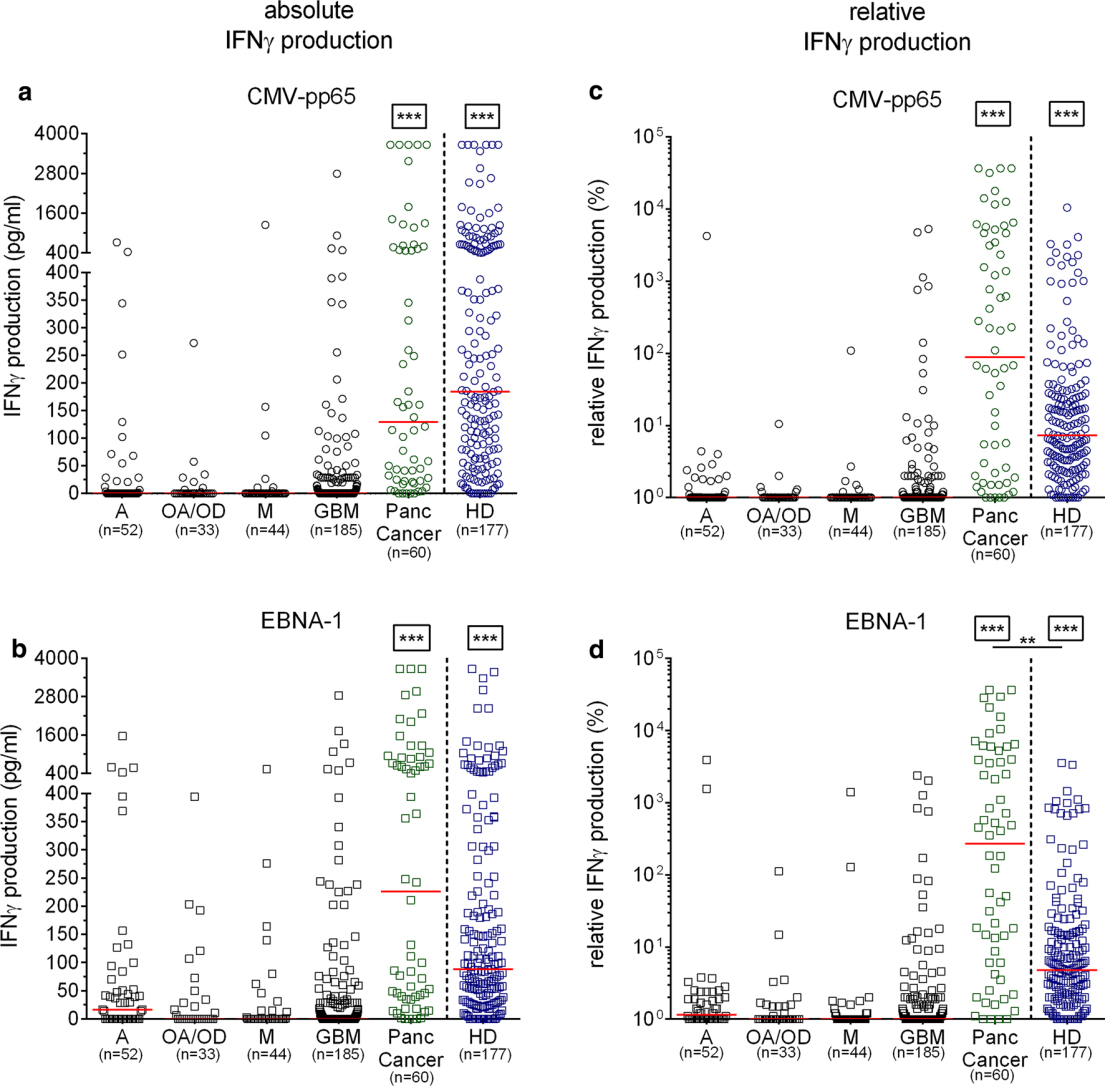
IFNγ production in response to EBV and CMV antigens in patients with cancer and in healthy donors. **a**, **b** Absolute values of the CMV-pp65 and EBNA-1-specific IFNγ production for each group. **c**, **d** Relative IFNγ production against CMV-pp65 and EBNA-1 antigens for each group. Kruskal–Wallis test followed by Dunn’s post-test was performed to gauge statistical significance *p < 0.05; **p < 0.01; ***p < 0.001. Box stars indicate statistical significance of IFNγ production with all the other groups. *GBM* glioblastoma multiforme, *A* astrocytoma, *OA/OD* oligoastrocytoma/oligodendroglioma, *M* metastatic disease, *Panc Cancer* pancreatic cancer and *HD* healthy donor

The whole blood assay (WBA) provides a quick and easy assessment of the cellular antigen-specific immune response of circulating lymphocytes since it can be performed directly in blood samples without the need for cell isolation. The response to the positive control (PHA) showed a significant lower IFNγ production by patients with brain tumor compared to patients with pancreatic cancer as well as HD (p < 0.001, Additional file 1: Figure S1A). Note that patients with pancreatic cancer produced significantly more IFNγ to PHA (p < 0.016) than HD while conversely, patients with pancreatic cancer exhibited significantly lower basal IFNγ production compared to patients with brain tumor as well as HD (p < 0.01, Additional file 1: Figure S1B).The basal IFNγ production was found to be higher in the patients with astrocytoma (A) as compared to HD and patients with pancreatic cancer (p < 0.001) as well with metastasis (p < 0.017). Regarding the response to viral antigens EBNA-1 and CMV-pp65, patients with brain tumor [GBM, OA/OD, astrocytoma (A) and brain metastasis (M)] produced significantly less IFNγ as compared to patients with pancreatic cancer as well as HD (p < 0.001, Fig. 2 a, b). No significant differences in the viral antigen specific IFNγ production among patients with brain cancer groups were observed.

We next examined whether differences in the antiviral cellular immune response between the patients groups and healthy donors were indeed specific to EBV and CMV antigens or rather a manifestation of disease-related immune status, considering that cancer treatment may also affect the patients’ immune response. This was particularly pertinent to patients with GBM, who receive heavy corticosteroid therapy (dexamethasone, betamethasone) following diagnosis for relief from the effects of non-specific, deleterious inflammation in the brain (largely oedema) while dampening their Th1 response [28]. The relative IFNγ production (hereafter referred to as rIFNγ) analysis allows normalization of the absolute cytokine concentration values measured and a more accurate assessment of the T cells’ capacity to respond to specific stimuli. Using this readout, we found patients with brain tumor to exhibit rIFNγ in response to CMV-pp65 and EBNA-1 significantly lower extent than HD and patients with pancreatic cancer (p < 0.001, Fig. 2 c, d) and only -EBNA-1-specific rIFNγ response of patients with pancreatic cancer was found higher as compared to HD (p = 0.006, Fig. 2 d).

In summary, patients with brain tumor are more likely to mount lower IFNγ responses in whole blood to both EBNA-1 and CMV-pp65 compared to healthy individuals and patients with pancreatic cancer.

### IFNγ responses of patients with brain tumor to EBV and CMV antigens with IL-2/IL-15/IL-21 conditioning

**Fig. 3:**
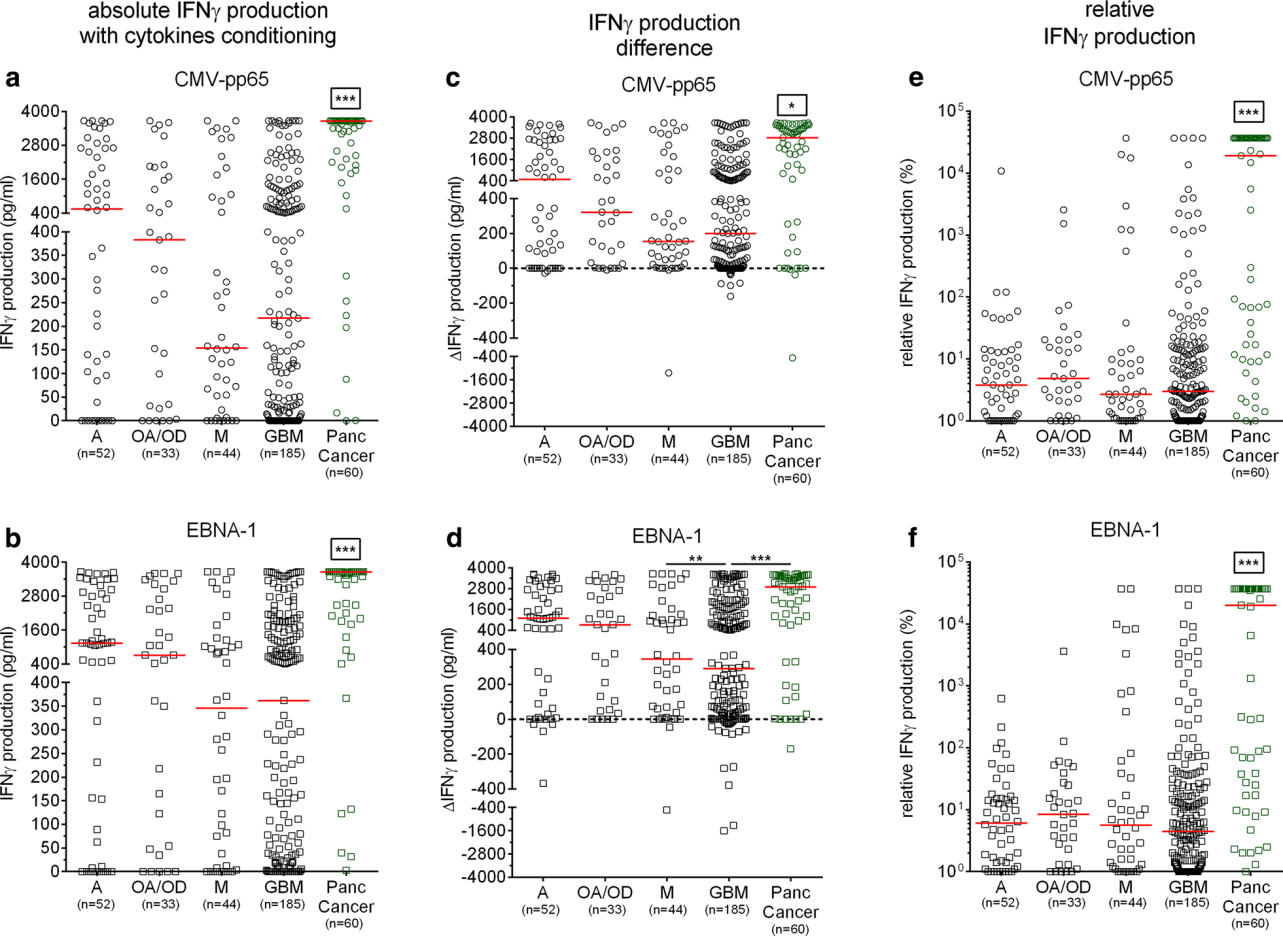
IFNγ production in response to EBV and CMV antigens under IL-2/IL-15/IL-21 cytokine conditioning in patients with cancer. **a**, **b** Absolute values of the CMV-pp65 and EBNA-1-specific IFNγ production for each group. **c**, **d** IFNγ production difference between unconditioned and with IL-2/IL-15/IL-21 cytokine conditioning in response to CMV-pp65 and EBNA-1 antigens. **e**, **f** Relative IFNγ production against CMV-pp65 and EBNA-1 antigens for each group. Kruskal–Wallis test followed by Dunn’s post-test was performed to gauge statistical significance *p < 0.05; **p < 0.01; ***p < 0.001. Box stars indicate statistical significance of IFNγ production with all the other groups. *GBM* glioblastoma multiforme, *A* astrocytoma, *OA/OD* oligoastrocytoma/oligodendroglioma, *M* metastatic disease and *Panc Cancer* pancreatic cancer

The whole blood of patients with cancer was also exposed to IL-2, IL-15 and IL-21 in culture, which we have previously reported to have a pronounced effect on amplifying tumor-directed T-cell responses [23, 29, 30]. Akin to our previous observation, patients with brain tumor mounted a significantly lower IFNγ response to EBNA-1 as well as CMV-pp65 compared to patients with pancreatic cancer (p < 0.001, Fig. 3 a, b). Differences observed between patients in the IFNγ response to controls (PHA and medium only) under cytokine conditioning was conserved and similar to the one observed without cytokine conditioning (Additional file 1: Figure S1A–D) In general, circulating lymphocytes from patients with cancer responded very well to cytokine conditioning, marked by an increase in IFNγ production (Fig. 3 a, b and Additional file 1: Figure S1C, D). Although, the immune reactivity to EBNA-1 and CMV-pp65 appeared to differ depending on the cancer type: the magnitude of increase in antiviral IFNγ response following cytokine conditioning of whole blood was less pronounced in patients with brain tumor compared to those with pancreatic cancer (p < 0.05, Fig. 3 c, d). Specifically, patients with GBM presented the lowest IFNγ increase production to CMV-pp65 and EBNA-1 (ΔIFNγ production median respectively only + 200 and + 291.2 pg/ml) that were significantly lower that the patients with pancreatic cancer (p < 0.001, ΔIFNγ production median up to + 2800 pg/ml, Fig. 3 c, d). Overall, the response to PHA stimulation after IL2/IL-15/IL-21 conditioning was significantly improved (Additional file 1: Figure S1E) while IFNγ production was not increased drastically in the absence of antigen stimulation (ΔIFNγ production median < + 100 pg/ml, Additional file 1: Figure S1F).

Under cytokine conditioning, the rIFNγ production generally showed relative improvement by a factor of > three-fold (Fig. 3 e, f) compared to the rIFNγ values without cytokine conditioning (Fig. 2 c, d). Similar to the observation made without cytokine conditioning, patients with brain tumor showed a significantly lower rIFNγ production as compared to the patients with pancreatic cancer (p < 0.001, Fig. 3 e, f).

In summary, patients with brain tumor are less responsive to cytokine stimulation as compared to patients with pancreatic cancer.

### Correlation between IFNγ response to EBV and CMV antigens among patients with brain tumor or pancreatic cancer

**Fig. 4:**
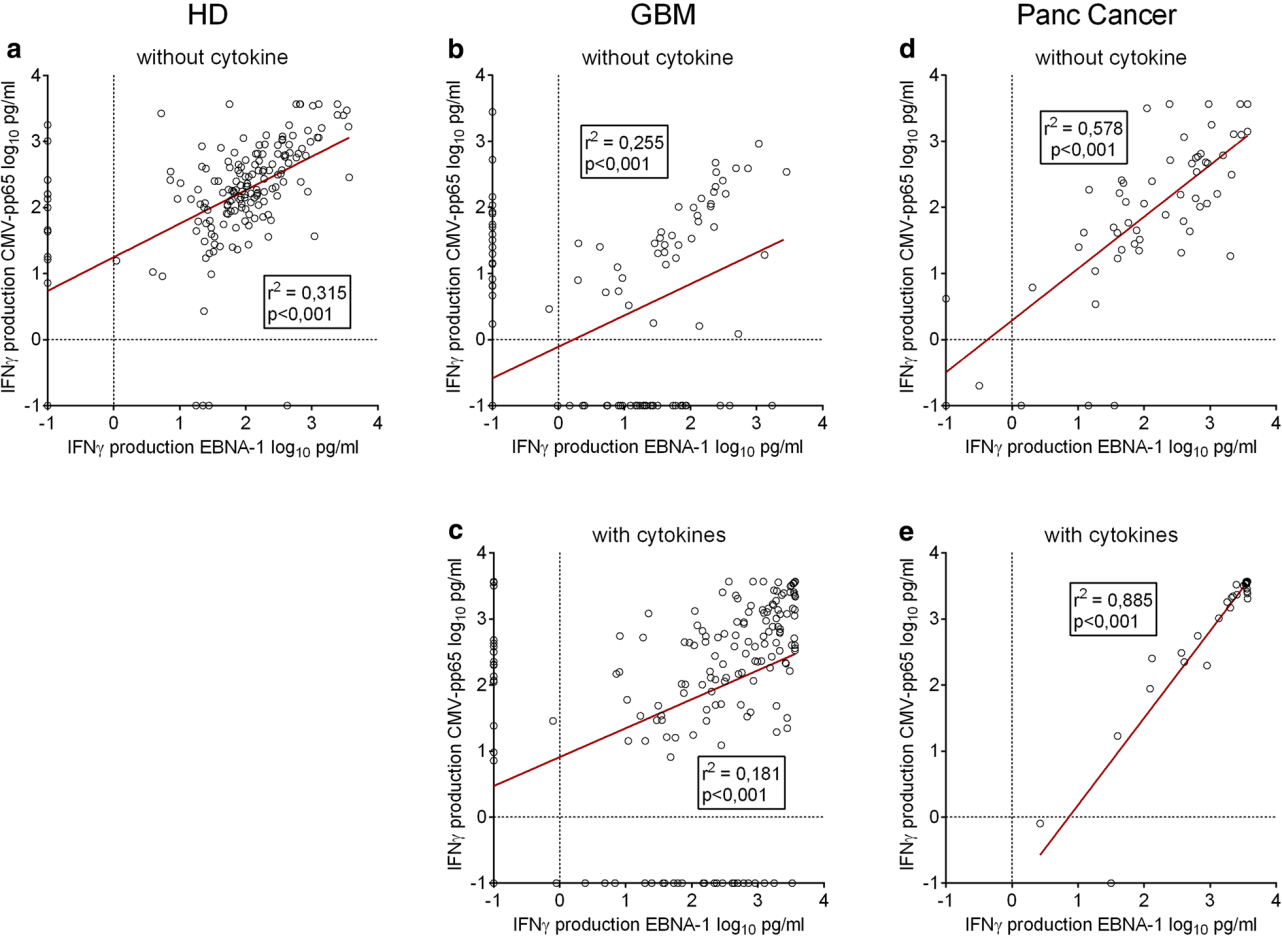
Correlation between EBNA-1 and CMV-pp65 immune responses among healthy individuals, patients with GBM and pancreatic cancer. **a** Correlation among healthy individuals. **b** Correlation among patients with brain tumor. **c** Correlation under IL-2/IL-15/IL-21 cytokine conditioning among patients with brain tumor. **d** Correlation among patients with pancreatic cancer. **e** Correlation under IL-2/IL-15/IL-21 cytokine conditioning among patients with pancreatic cancer. Linear regression was plotted, and a R^2^ value (steepness of curve) more than 0.5 was indicative of a direct correlation between EBV- and CMV-directed responses. *HD* healthy donor, *GBM* glioblastoma multiforme and *Panc Cancer* pancreatic cancer

In our analyses thus far, patients with GBM were immunologically more impaired than patients with pancreatic cancer in terms of cellular immune responses to the two studied viral antigens. To further analyse those observations, we test whether the immune responses of patients with cancer to EBNA-1 and CMV-pp65 were correlated to each other. Without cytokine conditioning, a poor correlation was observed among healthy individuals (r^2^ = 0.315, Fig. 4 a). The IFNγ responses to CMV and EBV in patients with GBM were not correlated in the absence (r^2^ = 0.255) or presence (r^2^ = 0.181) of cytokine conditioning (Fig. 4 b, c). Similar observation was made in the other patients with brain cancer (Additional file 1: Figure S1). Contrarily, patients with pancreatic cancer presented a significant correlation between immune reactivity to the viral antigens without cytokine conditioning (r^2^ = 0.578, Fig. 4 d) and cytokine conditioning showed to enhance that correlation (r^2^ = 0.885, Fig. 4 e**).**

Thus, we concluded that unlike patients with pancreatic cancer, patients with brain cancer did not shared direct correlation between their CMV- and EBV-specific cellular immune responses and did not have the correlation strengthened by cytokine conditioning.

### Survival of the patients with GBM correlates with IFNγ responses to EBV antigen

**Fig. 5:**
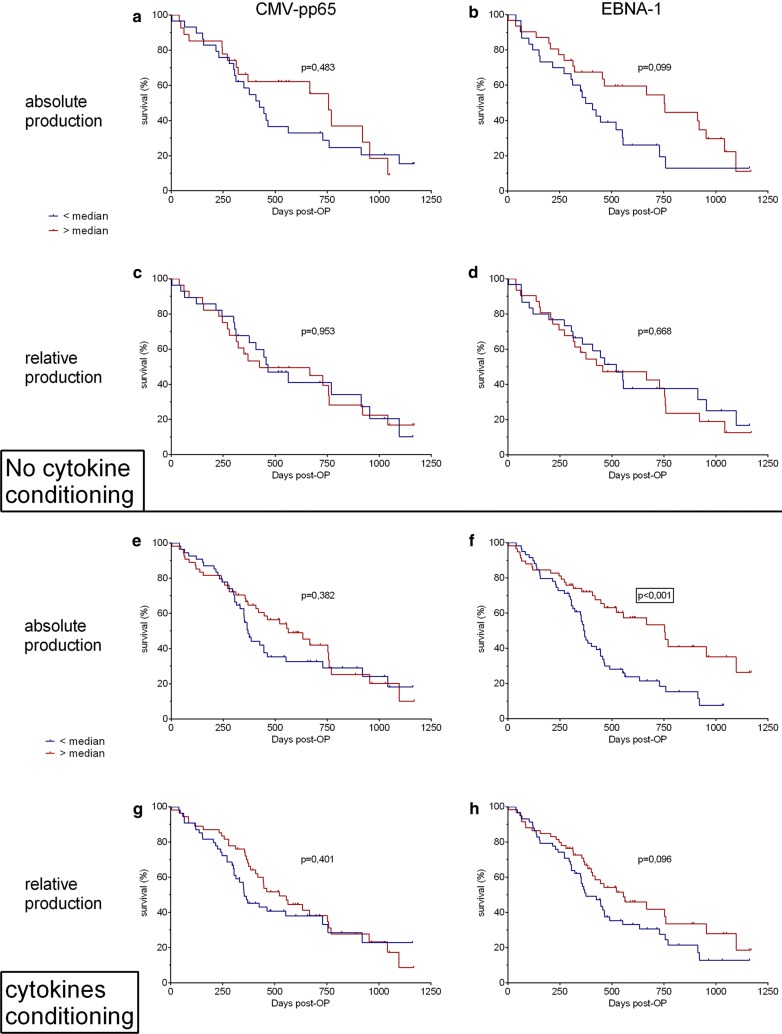
Survival of the patients with GBM based on the CMV- and EBV-specific IFNγ production. Kaplan–Meier curve shows the overall survival of GBM patients with detectable IFNγ response. Median concentration of detectable virus-specific IFNγ production was used as a cut-off to generate two separate groups: “< median” and “> median” antigen-specific response. **a–d** IFNγ production without IL-2/IL-15/IL-21 cytokine conditioning **a**, **b** Survival based on the absolute IFNγ production in response to CMV-pp65 (n = 56) and EBNA-1 (n = 61). **c, d** Survival based on the relative IFNγ production in response to CMV-pp65 (n = 56) and EBNA-1 (n = 61). **e–h** IFNγ production with IL-2/IL-15/IL-21 cytokine conditioning **e**, **f** Survival based on the absolute IFNγ production in response to CMV-pp65 (n = 108) and EBNA-1 (n = 117). **g**, **h** Survival based on the relative IFNγ production in response to CMV-pp65 (n = 108) and EBNA-1 (n = 117). *Post-OP* post-operation

Altogether, our data exposed an impaired cellular immune response of the patients with brain tumor and more specifically with GBM in contrast to healthy individuals or patients with pancreatic cancer. Considering the number of patients allocated to each group for the cellular immune response analysis, we were only able to evaluate the survival of patients with GBM due to the large cohort size (n = 136). Without cytokine conditioning, no correlation between survival post-surgery (post-OP) and the viral specific IFNγ production were defined (Fig. 5 a, b) but patients with an EBNA-1-specific IFNγ production above the median show a trend (p = 0.099) towards a better survival (median survival 753 versus 375 days). No difference in the survival was highlighted between patients with virus-specific rIFNγ production below and above the median (Fig. 5 c, d).

With cytokine conditioning, survival was not affected by the CMV-pp65-specific IFNγ production while the median survival post-surgery was higher among patients who responded robustly to EBNA-1 (p < 0.001, 753 versus 370 days) (Fig. 5 e, f). This observation however was also found to be true for PHA-driven IFNγ production (p = 0.025, Additional file 1: Figure S1). Therefore, to examine whether improved survival is a direct consequence of an efficient anti-viral response or rather a representation of the general immune potential of the patients reflected by their ability to respond to cytokine conditioning, we analyzed the survival pattern of the patients based on the rIFNγ values specific to EBNA-1 and CMV-pp65 antigens. While the rIFNγ to CMV-pp65 did not seem to influence the survival, this readout showed only a trend towards an improved overall median survival of the GBM patients with a robust EBNA-1-specific rIFNγ (555 vs 375 days p = 0.096, Fig. 5 g, h).

## Discussion

Chronic local inflammation in response to pathogens and/or autoantigens, orchestrated by a combination of innate and adaptive immune cell activity can lead to the oncogenesis of solid tumors [31]. Such perturbation of the overall immunological equilibrium may compromise the immune system’s ability to deter productive infection by latent pathogens and associated clinical disease [32, 33]. Therefore, well-preserved pathogen-directed immune responses reflect a certain level of immunological fitness in patients with cancer. IFNγ produced by T cells has, among its various biological functions, an essential role in the anti-viral and anti-tumor immune defense [34]. Earlier clinical studies involving patients with advanced cancer indicate that reduced or impaired IFNγ signaling may negatively affect patient survival [35–38]. The present report describes for the first time CMV- and EBV-specific immune responses—characterized by antigen-specific IFNγ production and humoral immune responses—in chemotherapy-naïve patients with pancreatic cancer or brain tumor.

First, we observed in the present study cohort that CMV-pp65-specific but not EBNA-1-specific plasma IgG levels were different among patients with cancer and healthy donors. Plasma IgG level is usually indicative of a past infection, and although not assayed in conjunction with other Ig subclasses recognizing EBV early antigen or viral capsid antigen, our data suggests that the patients in the cohort described here have well-preserved humoral immunity to EBV infection. On the contrary, patients with brain tumor showed a lower IgG response to CMV as opposed to patients with pancreatic cancer and healthy individuals. This observation agrees with a clinical study in which 8 out of 15 patients with high-grade glioma who presented with low-to-no CMV DNA present despite testing positive for CMV-specific IgG [13]. Earlier studies by Wrensch et al. [39, 40] described lower IgG response to varicella-zoster virus (VZV) but did not report any direct association with other herpesviruses i.e. CMV and EBV, while Amirian et al. [18] showed that among CMV IgG-positive patients, those with the lowest level of anti-CMV IgG presented the highest glioma risk. Of note, an exploratory study described the poor reliability of serology tests to define CMV infection in patients with GBM as the authors observed discrepancy between cellular and humoral immune reactivity to CMV peptides [19]. Additionally, CMV-specific T cells have been found at a low frequency yet protective against CMV infection in CMV-seronegative kidney transplant recipients and after a short CMV-pp65 peptide stimulation, 75% of healthy CMV-seronegative donors exhibited a CMV-specific T cell response [41, 42]. This discordant response between cellular and humoral immunity toward CMV antigens have been earlier described showing proliferative response to CMV antigens by PBMCs of CMV-seronegative donors [43]. Therefore EBV or CMV serology of healthy individuals or patients with cancer may not necessarily reflect the status of their cell-mediated immunity to these viruses.

Numerous studies and clinical observations have shown that immunological control of latent infection with CMV and/or EBV in humans requires adequately intact T-cell responses [9, 44, 45]. Thus, upon antigen encounter, these CMV-directed lymphocytes should be able to expand rapidly and respond with cytokine production (i.e. IFNγ, TNFα, IL-2) and/or cytotoxicity [46]. Interestingly, anti-CMV T-cell responses detected in human GBM tumor tissue have been suggested to play a role in driving anti-tumor immune responses in some patients [14, 47]. We however observed that exposure of peripheral blood of patients with malignant glioma to CMV-pp65 antigen in the absence of cytokine conditioning did not enhance significantly the IFNγ production. The same appeared to be true for exposure to EBNA-1 protein as well as PHA. It is noteworthy to point out here that patients with GBM or brain metastases displayed particularly meagre IFNγ responses to the viral proteins or mitogen. This is a stark representation of the general immune impairment in these individuals, which has been in part attributed to the reduced responsiveness of peripheral blood T cells from brain tumor patients to IL-2 further to the use of corticosteroids to reduce brain inflammation [28, 48]. Moreover, potentially elevated expression of immune checkpoint molecules i.e. programmed cell death 1 (PD-1), cytotoxic lymphocyte-associated antigen 4 (CTLA-4) could also drive general exhaustion of T cells in patients with GBM as previously reported [49, 50]. GBM is the most aggressive form of malignant glioma, while the occurrence of brain metastases is also suggested to impair cell-mediated immunity owing to increased transforming growth factor beta (TGF-β) levels, elevated numbers of circulating and tumor-infiltrating regulatory T cells (Treg) and non-productive inflammatory processes [3, 28, 51]. Also, CMV- and EBV-directed IFNγ production in peripheral blood has been linked to better survival among patients with tuberculosis (characterized by aberrant immunopathology leading to systemic immune dysregulation that also manifests in T-cell exhaustion) after successful completion of standard antibiotic therapy [52]. Thus, albeit immune checkpoint expression on T cells, their ability to react to stimulation by CMV/EBV is not abrogated and thus hints at a more intense nature of immune suppression experienced by patients with glioma compared to those with pancreatic cancer.

Impairment of T-cell activity due to cellular exhaustion (where inflammation plays an important role) can generally predict disease progression, thus patient survival. Since immune exhaustion is characterized by reduced ability to produce cytokines and proliferate, the IFNγ production profile in whole blood presented represents an important characteristic of immune fitness—with regard to the cellular immune compartment [53, 54]. EBV and CMV specific T cells can have high PD-1 expression, resulting from persistent TCR stimulation during chronic infection [55], which is also governed by T-cell memory and activation status thus exerting a profound effect on their functionality [56, 57]. Interestingly, CMV-specific T cells have also been implicated in clearance of chronic hepatitis C virus infection [58], while being functionally intact in patients with chronic lymphocytic leukemia despite general T-cell impairment in addition to immune exhaustion [59]. Also, CMV- and EBV-directed IFNγ production in peripheral blood has been linked to better survival among patients with tuberculosis (characterized by chronic inflammation) after successful completion of standard antibiotic therapy [52].

Cytokine conditioning of peripheral blood in the present study had a pronounced effect on IFNγ production to viral antigen exposure. Furthermore, the influence of the cytokine conditioning-induced increase of IFNγ production in whole blood appeared to vary between patient groups and the viral antigens themselves (CMV-pp65 vs EBNA-1). T cell populations may display different degrees of sensitivity to cytokine exposure largely due to immune dysfunction-driven dynamics of the surface cytokine receptor expression, as reported in cancer [48]. Furthermore, we previously reported that TILs from pancreatic tumor as well as GBM tissue can be successfully expanded in culture medium containing IL-2, IL-15 and IL-21, leading to proliferation of central memory T cells with strong effector functions and a rich TCRVβ repertoire, hinting at a wide epitope recognition potential [29, 30]. In addition, cytokine effect in reducing the numbers of pre-apoptotic cells and restoring TCR function in patients with HIV infection or acute myeloid leukemia has been previously reported [60, 61]. In agreement with the existing evidence, treating whole blood of patients with cancer with IL-2, IL-15 and IL-21 markedly improved the antigen-specific IFNγ response regardless of existing immunosuppression, thereby suggesting clinical applicability.

Recognition of CMV antigens by plasma IgG in patients with GBM is generally lower as compared to healthy individuals and patients with pancreatic cancer, although CMV IgG-positive patients with GBM with a higher antibody titer do significantly better from a clinical perspective (pertaining to survival after surgery). We have shown that compared to patients with pancreatic cancer and healthy individuals, immune function represented by CMV- and EBV-specific cellular response of patients with brain tumor is impaired. This immune reactivity may be improved by cytokine conditioning of lymphocytes. Also, IFNγ production to EBV was found to be of interest in patients with GBM. This correlation was only observed with IL-2/IL-15/IL-21 conditioning of the peripheral blood affirming that our finding are not only related to the specific immune response to EBV an antigen but more likely also reflecting the immune fitness of the patient with GBM as the capability to respond to cytokines stimulation.

We acknowledge that this study is limited the investigation to the anti-viral immune response characterized by the CMV-specific IgG level and the IFNγ production by circulating immune cells in blood in patients with brain cancer in comparison with those with pancreatic cancer. While our results show that the competence of the general and CMV/EBV specific immune response impact on clinical outcome, we acknowledge that a myriad of other clinical parameters govern patient survival as were shown in a previous publication from our laboratory concerning the predictability of the survival of patients with brain metastases based on anti-mesothelin immune responses in blood shown by multivariate analysis [62]. Nevertheless, this is the first report to show link between immune response patterns to CMV and EBV in relation to survival of patients with brain tumor, and be developed for future studies with the inclusion of necessary clinical criteria [63, 64].

## Conclusions

Antiviral immune responses, especially to common pathogens such as CMV and EBV are an integral component of immune competence in humans. These findings shed new light on our understanding of antiviral immune responses in patients with brain tumor while reflecting the general immunological status of patients with cancer in response to T cell-oriented cytokines and/or common viral antigens. Thus, efforts to better understand and characterize the cellular exhaustion/impairment caused by persistent chronic infection as well as the tumor microenvironment may lead to novel strategies for exploiting functional and clinically relevant immune responses for therapy.

## Additional files

**Additional file 1: Figure S1.** IFNγ production in response to positive control (PHA) and negative control (medium, no antigen stimulation) in patients with cancer and in healthy donors. **A-B.** Absolute values of the PHA-specific and basal (medium) IFNγ production for each group. **C-D.** Absolute values of the PHA-specific and basal (medium) IFNγ production for each group under IL-2/IL-15/IL-21 cytokine conditioning. **E-F.** IFNγ production difference between unconditioned and with IL-2/IL-15/IL-21 cytokine conditioning. Kruskal–Wallis test followed by Dunn’s post-test was performed to gauge statistical significance *p<0.05; **p<0.01; ***p<0.001. Box stars indicate statistical significance of IFNγ production with all the other groups. GBM: Glioblastoma multiforme, A: astrocytoma, OA/OD: oligoastrocytoma/ oligodendroglioma, M: metastatic disease and Panc Cancer: pancreatic cancer.

**Additional file 2: Figure S1** Correlation between EBNA-1 and CMV-pp65 immune responses among patients with brain tumor. **A-B.** Correlation among patients with astrocytoma without and with cytokine conditioning. **C-D.** Correlation among patients with oligoastrocytoma/ oligodendroglioma without and with cytokine conditioning. **E-F.** Correlation among patients with brain metastasis without and with cytokine conditioning. Linear regression was plotted, and a R^2^ value (steepness of curve) more than 0.5 was indicative of a direct correlation between EBV- and CMV-directed responses.

**Additional file 3: Figure S1** Survival of the patients with GBM based on antigen-specific IFNγ production. Kaplan-Meier curve shows the overall survival of GBM patients with detectable IFNγ response. Median concentration of detectable virus-specific IFNγ production was used as a cut-off to generate two separate groups: “< median” and “> median” antigen-specific response. **A-B.** Survival of the patients with GBM based on the absolute PHA-specific (n=110) and basal (n=108) IFNγ production without cytokine conditioning. **C-D.** Survival of the patients with GBM based on the absolute PHA-specific (n=133) and basal (n=118) IFNγ production with IL-2/IL-15-IL21 conditioning. Post-OP: post-operation.

## Abbreviations

A: astrocytoma
ADCC: antibody-dependent cellular cytotoxicity
CD: cluster of differentiation
CMV: cytomegalovirus
CMV-pp65: cytomegalovirus structural protein pp65
EBV: Epstein–Barr virus
EBNA-1: Epstein–Barr nuclear antigen-1
ELISA: enzyme-linked immunosorbent assay
GBM: glioblastoma multiforme
HD: healthy donor
HIV: human immunodeficiency virus
IFNγ: interferon gamma
IgG: immunoglobulin G
IL: interleukin
M: metastatic disease
NK: natural killer cells
OA/OD: oligoastrocytoma/oligodendroglioma
PBMCs: peripheral blood mononuclear cells
Post-OP: post-operation
PTLD: post-transplant lymphoproliferative disorder
TCR: T-cell receptor
TIL: tumor infiltrating lymphocytes
TNFα: tumor necrosis factor alpha
VZV: varicella-zoster virus
WBA: whole blood assay

## References

[1] Stewart B, Wild C, WHO (2014). World Cancer Report 2014. Lyon: International Agency for Research on Cancer.

[2] Kunk PR, Bauer TW, Slingluff CL, Rahma OE (2016). From bench to bedside a comprehensive review of pancreatic cancer immunotherapy. J Immunother Cancer.

[3] Maher EA, Furnari FB, Bachoo RM, Rowitch DH, Louis DN, Cavenee WK, DePinho RA (2001). Malignant glioma: genetics and biology of a grave matter. Genes Dev.

[4] Protti MP, De Monte L (2013). Immune infiltrates as predictive markers of survival in pancreatic cancer patients. Front Physiol.

[5] Baniyash M (2004). TCR zeta-chain downregulation: curtailing an excessive inflammatory immune response. Nat Rev Immunol.

[6] Quinn M, Erkes DA, Snyder CM (2016). Cytomegalovirus and immunotherapy: opportunistic pathogen, novel target for cancer and a promising vaccine vector. Immunotherapy.

[7] Khawcharoenporn T, Lau WK, Chokrungvaranon N (2008). Epstein–Barr virus infection with acute pancreatitis. Int J Infect Dis.

[8] Lopez-Verges S, Milush JM, Pandey S, York VA, Arakawa-Hoyt J, Pircher H, Norris PJ, Nixon DF, Lanier LL (2010). CD57 defines a functionally distinct population of mature NK cells in the human CD56dimCD16+ NK-cell subset. Blood.

[9] Marrao G, Habib M, Paiva A, Bicout D, Fallecker C, Franco S, Fafi-Kremer S, Simoes da Silva T, Morand P, Freire de Oliveira C (2014). Epstein–Barr virus infection and clinical outcome in breast cancer patients correlate with immune cell TNF-alpha/IFN-gamma response. BMC Cancer.

[10] Akhtar S, Vranic S, Cyprian FS, Al Moustafa AE (2018). Epstein–Barr virus in gliomas: cause, association, or artifact?. Front Oncol.

[11] Wick W, Platten M (2014). CMV infection and glioma, a highly controversial concept struggling in the clinical arena. Neuro-oncology.

[12] Stragliotto G, Rahbar A, Solberg NW, Lilja A, Taher C, Orrego A, Bjurman B, Tammik C, Skarman P, Peredo I (2013). Effects of valganciclovir as an add-on therapy in patients with cytomegalovirus-positive glioblastoma: a randomized, double-blind, hypothesis-generating study. Int J Cancer.

[13] Holdhoff M, Guner G, Rodriguez FJ, Hicks JL, Zheng Q, Forman MS, Ye X, Grossman SA, Meeker AK, Heaphy CM (2017). Absence of cytomegalovirus in glioblastoma and other high-grade gliomas by real-time PCR, immunohistochemistry, and in situ hybridization. Clin Cancer Res.

[14] Nair SK, De Leon G, Boczkowski D, Schmittling R, Xie W, Staats J, Liu R, Johnson LA, Weinhold K, Archer GE (2014). Recognition and killing of autologous, primary glioblastoma tumor cells by human cytomegalovirus pp65-specific cytotoxic T cells. Clin Cancer Res.

[15] Pfirrmann V, Oelsner S, Rettinger E, Huenecke S, Bonig H, Merker M, Wels WS, Cinatl J, Schubert R, Klingebiel T (2015). Cytomegalovirus-specific cytokine-induced killer cells: concurrent targeting of leukemia and cytomegalovirus. Cytotherapy.

[16] Batich KA, Reap EA, Archer GE, Sanchez-Perez L, Nair SK, Schmittling RJ, Norberg P, Xie W, Herndon JE, Healy P (2017). Long-term survival in glioblastoma with cytomegalovirus pp65-targeted vaccination. Clin Cancer Res.

[17] Sjostrom S, Hjalmars U, Juto P, Wadell G, Hallmans G, Tjonneland A, Halkjaer J, Manjer J, Almquist M, Melin BS (2011). Human immunoglobulin G levels of viruses and associated glioma risk. Cancer Causes Control (CCC).

[18] Amirian ES, Marquez-Do D, Bondy ML, Scheurer ME (2013). Anti-human-cytomegalovirus immunoglobulin G levels in glioma risk and prognosis. Cancer Med.

[19] Rahbar A, Peredo I, Solberg NW, Taher C, Dzabic M, Xu X, Skarman P, Fornara O, Tammik C, Yaiw K (2015). Discordant humoral and cellular immune responses to Cytomegalovirus (CMV) in glioblastoma patients whose tumors are positive for CMV. Oncoimmunology.

[20] Hochberg D, Middeldorp JM, Catalina M, Sullivan JL, Luzuriaga K, Thorley-Lawson DA (2004). Demonstration of the Burkitt’s lymphoma Epstein–Barr virus phenotype in dividing latently infected memory cells in vivo. Proc Natl Acad Sci USA.

[21] Olsson J, Kok E, Adolfsson R, Lovheim H, Elgh F (2017). Herpes virus seroepidemiology in the adult Swedish population. Immun Ageing.

[22] Andersson-Ellstrom A, Svennerholm B, Forssman L (1995). Prevalence of antibodies to herpes simplex virus types 1 and 2, Epstein–Barr virus and cytomegalovirus in teenage girls. Scand J Infect Dis.

[23] Liu Z, Poiret T, Persson C, Meng Q, Rane L, Bartek J, Karbach J, Altmannsberger HM, Illies C, Luo X (2018). NY-ESO-1- and survivin-specific T-cell responses in the peripheral blood from patients with glioma. Cancer Immunol Immunother.

[24] Magalhaes I, Eriksson M, Linde C, Muhammad R, Rane L, Ambati A, Axelsson-Robertson R, Khalaj B, Alvarez-Corrales N, Lapini G (2014). Difference in immune response in vaccinated and unvaccinated Swedish individuals after the 2009 influenza pandemic. BMC Infect Dis.

[25] Ambati A, Boas LS, Ljungman P, Testa L, de Oliveira JF, Aoun M, Colturato V, Maeurer M, Machado CM (2015). Evaluation of pretransplant influenza vaccination in hematopoietic SCT: a randomized prospective study. Bone Marrow Transplant.

[26] Lagrelius M, Jones P, Franck K, Gaines H (2006). Cytokine detection by multiplex technology useful for assessing antigen specific cytokine profiles and kinetics in whole blood cultured up to seven days. Cytokine.

[27] Alvarez-Corrales N, Ahmed RK, Rodriguez CA, Balaji KN, Rivera R, Sompallae R, Vudattu NK, Hoffner SE, Zumla A, Pineda-Garcia L (2013). Differential cellular recognition pattern to *M. tuberculosis* targets defined by IFN-gamma and IL-17 production in blood from TB+ patients from Honduras as compared to health care workers: TB and immune responses in patients from Honduras. BMC Infect Dis.

[28] Dietrich J, Rao K, Pastorino S, Kesari S (2011). Corticosteroids in brain cancer patients: benefits and pitfalls. Expert Rev Clin Pharmacol.

[29] Meng Q, Liu Z, Rangelova E, Poiret T, Ambati A, Rane L, Xie S, Verbeke C, Dodoo E, Del Chiaro M (2016). Expansion of tumor-reactive T cells from patients with pancreatic cancer. J Immunother.

[30] Liu Z, Meng Q, Bartek J, Poiret T, Persson O, Rane L, Rangelova E, Illies C, Peredo IH, Luo X (2017). Tumor-infiltrating lymphocytes (TILs) from patients with glioma. Oncoimmunology.

[31] Crusz SM, Balkwill FR (2015). Inflammation and cancer: advances and new agents. Nat Rev Clin Oncol.

[32] Karnak D, Kayacan O, Beder S (2002). Reactivation of pulmonary tuberculosis in malignancy. Tumori.

[33] Schlick K, Grundbichler M, Auberger J, Kern JM, Hell M, Hohla F, Hopfinger G, Greil R (2015). Cytomegalovirus reactivation and its clinical impact in patients with solid tumors. Infect Agents Cancer.

[34] Schroder K, Hertzog PJ, Ravasi T, Hume DA (2004). Interferon-gamma: an overview of signals, mechanisms and functions. J Leukoc Biol.

[35] Lee IC, Huang YH, Chau GY, Huo TI, Su CW, Wu JC, Lin HC (2013). Serum interferon gamma level predicts recurrence in hepatocellular carcinoma patients after curative treatments. Int J Cancer.

[36] Critchley-Thorne RJ, Simons DL, Yan N, Miyahira AK, Dirbas FM, Johnson DL, Swetter SM, Carlson RW, Fisher GA, Koong A (2009). Impaired interferon signaling is a common immune defect in human cancer. Proc Natl Acad Sci USA.

[37] Critchley-Thorne RJ, Yan N, Nacu S, Weber J, Holmes SP, Lee PP (2007). Down-regulation of the interferon signaling pathway in T lymphocytes from patients with metastatic melanoma. PLoS Med.

[38] Martin F, Santolaria F, Batista N, Milena A, Gonzalez-Reimers E, Brito MJ, Oramas J (1999). Cytokine levels (IL-6 and IFN-gamma), acute phase response and nutritional status as prognostic factors in lung cancer. Cytokine.

[39] Wrensch M, Weinberg A, Wiencke J, Masters H, Miike R, Barger G, Lee M (1997). Does prior infection with varicella-zoster virus influence risk of adult glioma?. Am J Epidemiol.

[40] Wrensch M, Weinberg A, Wiencke J, Miike R, Barger G, Kelsey K (2001). Prevalence of antibodies to four herpesviruses among adults with glioma and controls. Am J Epidemiol.

[41] Litjens NHR, Huang L, Dedeoglu B, Meijers RWJ, Kwekkeboom J, Betjes MGH (2017). Protective cytomegalovirus (CMV)-specific T-cell immunity is frequent in kidney transplant patients without serum anti-CMV antibodies. Front Immunol.

[42] Loeth N, Assing K, Madsen HO, Vindelov L, Buus S, Stryhn A (2012). Humoral and cellular CMV responses in healthy donors; identification of a frequent population of CMV-specific, CD4+ T cells in seronegative donors. PLoS ONE.

[43] Zhu J, Shearer GM, Marincola FM, Norman JE, Rott D, Zou JP, Epstein SE (2001). Discordant cellular and humoral immune responses to cytomegalovirus infection in healthy blood donors: existence of a Th1-type dominant response. Int Immunol.

[44] Cohen JI (2000). Epstein–Barr virus infection. N Eng J Med.

[45] La Rosa C, Diamond DJ (2012). The immune response to human CMV. Future Virol.

[46] Klenerman P, Oxenius A (2016). T cell responses to cytomegalovirus. Nat Rev Immunol.

[47] Michaelis M, Doerr HW, Cinatl J (2009). The story of human cytomegalovirus and cancer: increasing evidence and open questions. Neoplasia.

[48] Dix AR, Brooks WH, Roszman TL, Morford LA (1999). Immune defects observed in patients with primary malignant brain tumors. J Neuroimmunol.

[49] Wei B, Wang L, Zhao X, Du C, Guo Y, Sun Z (2014). The upregulation of programmed death 1 on peripheral blood T cells of glioma is correlated with disease progression. Tumour Biol.

[50] Mirzaei R, Sarkar S, Yong VW (2017). T cell exhaustion in glioblastoma: intricacies of immune checkpoints. Trends Immunol.

[51] Lowther DE, Goods BA, Lucca LE, Lerner BA, Raddassi K, van Dijk D, Hernandez AL, Duan X, Gunel M, Coric V (2016). PD-1 marks dysfunctional regulatory T cells in malignant gliomas. JCI insight.

[52] Nagu T, Aboud S, Rao M, Matee M, Axelsson R, Valentini D, Mugusi F, Zumla A, Maeurer M (2017). Strong anti-Epstein Barr virus (EBV) or cytomegalovirus (CMV) cellular immune responses predict survival and a favourable response to anti-tuberculosis therapy. Int J Infect Dis (IJID).

[53] Wherry EJ, Blattman JN, Murali-Krishna K, van der Most R, Ahmed R (2003). Viral persistence alters CD8 T-cell immunodominance and tissue distribution and results in distinct stages of functional impairment. J Virol.

[54] Zajac AJ, Blattman JN, Murali-Krishna K, Sourdive DJ, Suresh M, Altman JD, Ahmed R (1998). Viral immune evasion due to persistence of activated T cells without effector function. J Exp Med.

[55] Youngblood B, Oestreich KJ, Ha SJ, Duraiswamy J, Akondy RS, West EE, Wei Z, Lu P, Austin JW, Riley JL (2011). Chronic virus infection enforces demethylation of the locus that encodes PD-1 in antigen-specific CD8(+) T cells. Immunity.

[56] Legat A, Speiser DE, Pircher H, Zehn D, Fuertes Marraco SA (2013). Inhibitory receptor expression depends more dominantly on differentiation and activation than “exhaustion” of human CD8 T cells. Front Immunol.

[57] Duraiswamy J, Ibegbu CC, Masopust D, Miller JD, Araki K, Doho GH, Tata P, Gupta S, Zilliox MJ, Nakaya HI (2011). Phenotype, function, and gene expression profiles of programmed death-1(hi) CD8 T cells in healthy human adults. J Immunol.

[58] Owusu Sekyere S, Suneetha PV, Hardtke S, Falk CS, Hengst J, Manns MP, Cornberg M, Wedemeyer H, Schlaphoff V (2015). Type I interferon elevates co-regulatory receptor expression on CMV-and EBV-specific CD8 T Cells in chronic hepatitis C. Front Immunol.

[59] te Raa GD, Pascutti MF, Garcia-Vallejo JJ, Reinen E, Remmerswaal EB, ten Berge IJ, van Lier RA, Eldering E, van Oers MH, Tonino SH (2014). CMV-specific CD8+ T-cell function is not impaired in chronic lymphocytic leukemia. Blood.

[60] Patterson J, Jesser R, Weinberg A (2008). Distinctive in vitro effects of T-cell growth cytokines on cytomegalovirus-stimulated T-cell responses of HIV-infected HAART recipients. Virology.

[61] Shi L, Chen S, Zha X, Xu Y, Xu L, Yang L, Lu Y, Zhu K, Li Y (2015). Enhancement of the TCRzeta expression, polyclonal expansion, and activation of t cells from patients with acute myeloid leukemia after IL-2, IL-7, and IL-12 induction. DNA Cell Biol.

[62] Zhenjiang L, Rao M, Luo X, Sandberg E, Bartek J, Schoutrop E, von Landenberg A, Meng Q, Valentini D, Poiret T (2017). Mesothelin-specific immune responses predict survival of patients with brain metastasis. EBioMedicine.

[63] Carson KA, Grossman SA, Fisher JD, Shaw EG (2007). Prognostic factors for survival in adult patients with recurrent glioma enrolled onto the new approaches to brain tumor therapy CNS consortium phase I and II clinical trials. J clin Oncol.

[64] Stewart LA (2002). Chemotherapy in adult high-grade glioma: a systematic review and meta-analysis of individual patient data from 12 randomised trials. Lancet.

